# Partial breast irradiation with CyberKnife after breast conserving surgery: a pilot study in early breast cancer

**DOI:** 10.1186/s13014-018-0991-4

**Published:** 2018-03-23

**Authors:** Laura Lozza, Laura Fariselli, Marco Sandri, Mario Rampa, Valentina Pinzi, Maria Carmen De Santis, Marzia Franceschini, Giovanna Trecate, Ilaria Maugeri, Luisa Fumagalli, Francesca Bonfantini, Giulia Bianchi, Emanuele Pignoli, Elena De Martin, Roberto Agresti

**Affiliations:** 1 Radiotherapy Unit, Fondazione IRCCS Istituto Nazionale dei Tumori, Milan, Italy; 2 Health Department, Fondazione IRCCS Istituto Neurologico Carlo Besta, Milan, Italy; 3 Molecular Targeting Unit, Fondazione IRCCS Istituto Nazionale dei Tumori, Milan, Italy; 4 Breast Surgery Unit, Fondazione IRCCS Istituto Nazionale dei Tumori, Milan, Italy; 5 Radiology Unit, Fondazione IRCCS Istituto Nazionale dei Tumori, Milan, Italy; 6 Medical Physics Unit, Fondazione IRCCS Istituto Nazionale dei Tumori, Milan, Italy; 7 Medical Oncology Unit, Fondazione IRCCS Istituto Nazionale dei Tumori, Milan, Italy

## Abstract

**Background:**

Local recurrences after breast conserving treatment are mainly close to the original tumor site, and as such shorter fractionation strategies focused on and nearest mammary gland, i.e. accelerated partial breast irradiation (APBI), have been developed. Stereotactic APBI has been attempted, although there is little experience using CyberKnife (CK) for early breast cancer.

**Methods:**

This pilot study was designed to assess the feasibility of CK-APBI on 20 evaluable patients of 29 eligible, followed for 2 years. The primary endpoint was acute/sub-acute toxicity; secondary endpoints were late toxicity and the cosmetic result.

**Results:**

Mean pathological tumor size was 10.5 mm (±4.3, range 3–18), 8 of these patients were classified as LumA-like, 11 as LumB-like, and 1 as LumB-HER2-enriched.

Using CK-APBI with Iris, the treatment time was approximately 60 min (range~ 35 to ~ 120). All patients received 30 Gy in five fractions delivered to the PTV. The median number of beams was 180 (IQR 107–213; range:56–325) with a median PTV isodose prescription of 86.0% (IQR 85.0–88.5; range:82–94). The median PTV was 88.1 cm3 (IQR 63.8–108.6; range:32.3–238.8). The median breast V100 and V50 was 0.6 (IQR 0.1–1.5; range:0–13) and 18.6 (IQR 13.1–21.7; range:7.5–37), respectively. The median PTV minimum dose was 26.2 Gy (IQR 24.7–27.6; range 22.3–29.3). Mild side effects were recorded during the period of observation. Cosmetic evaluations were performed by three observers from the start of radiotherapy up to 2 years. Patients’ evaluation progressively increase from 60% to 85% of excellent rating; this trend was similar to that of external observer.

**Conclusions:**

These preliminary results showed the safe feasibility of CK-APBI in early breast cancer, with mild acute and late toxicity and very good cosmetic results.

**Trial registration:**

The present study is registered at Clinicaltrial.gov (NCT02896322). Retrospectively egistered August 4, 2016.

## Background

Breast-conserving surgery (BCS) followed by post-surgical whole breast radiotherapy (WBRT), generally delivered in 5–6 weeks, has become the standard of care of early breast cancer during the last decades [1–3].

However, conventional WBRT may be associated with logistical difficulties considering the long time needed [4–6], short- and long-term toxicities, and poor cosmetic outcomes [6–9].

Recently, a standardized approach delivering the same radiotherapy to all patients no longer seems suitable considering a modern vision of personalized treatments for different biological and bio-molecular subtypes of breast cancer.

Furthermore, the large majority of local recurrences after BCS are close to the original tumor site, generally within 2 cm of surgical margins [10, 11], suggesting that it is possible to restrict the radiotherapy target to the surgical cavity in selected patients at low risk of recurrence; in this case, a much smaller volume than the whole breast has to be irradiated, and a more intense course of radiotherapy with fewer and larger dose fractions must be used. Consequently, shorter fractionation strategies focusing only on the tumor bed with a small cuff of surrounding subclinical disease, i.e. partial breast irradiation (PBI) or accelerated partial breast irradiation (APBI), have been developed as an alternative to WBRT [12–14]. This strategy is further supported by findings from studies using a reduced number of fractions (hypofractionation) [15].

APBI has been evaluated in several clinical trials [16–19] and technical approaches, including both external beam [20, 21] and brachytherapy [22–24], most commonly using 10 fractions over a 1 week period.

Another variation of APBI is intraoperative radiotherapy (IORT) or Electron Intra Operative Radiation Therapy (IOERT) [25], where the tumor bed is irradiated with low energy X-rays or electrons prior to closure of the surgical cavity, although pre-surgical selection of patients is sub-optimal.

To addressing the concerns of WBRT and limitations of IORT, stereotactic accelerated partial breast irradiation delivered with a CyberKnife radiosurgery system (CK-APBI) was recently introduced for early breast cancer and consists of a short course, focused on the target tissue. This technique is supposed to improve the accuracy of treatment, due to real-time tracking and respiratory motion control that reduce radiation delivery uncertainty with maximal target coverage. On the basis of these considerations, we speculate that it could be a convenient alternative compared to already known PBI techniques.

Although CK-APBI may be a highly innovative and interesting approach for early breast cancer, there is very little experience to date [26–30]. In fact, the mobility and pliability of breast tissue, making it a more difficult region to apply stereotactic localization than the brain or thorax, may represent a challenge.

In our pilot study, a series of 20 evaluable patients is presented to assess the feasibility and safety of CK-APBI in early breast cancer.

## Methods

This pilot phase I prospective study was designed to assess the feasibility of CK-APBI in low risk early breast cancer patients after BCS. Eligibility of patients and exclusion criteria are summarized in Table 1.

**Table 1:** Eligibility and exclusion criteria of the study

Inclusion criteria
- Age ≥ 45 years
- Stage I-IIA histologically confirmed breast carcinoma
- Tumor-free (at least 2 mm or more) inked histologic margins at surgical resection.
Clinical exclusion criteria
- Pregnancy
- Collagen vascular disease
- Aesthetic additive prostheses
- Severe cardiac, pulmonary and liver diseases
- Psychiatric illness compromising the correct acquisition of informed consent
Oncologic exclusion criteria
- Invasive lobular or multicentric carcinoma
- Extensive associated non-invasive ductal carcinoma (synchronous or previous)
- Peritumoral vascular invasion (> 3 vessels)
- BRCA mutation carriers
- More than 4 involved axillary lymph nodes
- Distant metastasis
- Non-epithelial malignancies of the breast
- Synchronous contralateral invasive carcinoma
- Paget disease, history of previous malignancy (except non-melanoma skin cancers and in situ oral cavity and cervix carcinoma)

This study was approved by Ethic Committee of both involved institutes and written informed consent was signed by each patient enrolled. The present study is registered at *Clinicaltrial.gov* (NCT02896322).

### Primary and secondary endpoints of the study

Assessing the feasibility of the CK-APBI, the primary endpoint was evaluation of acute and the sub-acute toxicity. Secondary endpoints were late toxicity and the cosmetic result.

### Preoperative radiologic evaluation

All patients underwent bilateral mammography and breast ultrasound (US). A pre-operative bilateral breast magnetic resonance imaging (MRI) was required to evaluate the extent of tumor, multicentric disease, and contralateral breast.

### Breast conserving surgery and gold seed positioning

BCS performed was formally defined as quadrantectomy and previously described [2]. Actually, in early breast cancer, BCS consists of partial removal of the breast gland surrounding the primary tumor, with the narrowest margin of at least at 1 cm, without removing the muscular fascia. In all cases, resection margins were specifically evaluated to be free of invasive or in-situ carcinoma.

During surgical intervention, after removal of the breast tumor, 2 mm gold seeds used as fiducial markers for driving CK-APBI were positioned around the surgical cavity after the breast glandular reshape and before suture of the surgical wound. These gold seeds were conventionally positioned on three different spatial planes, at the three corners of a ideal triangle centered on the area in which the tumor was removed. The first gold seed was put in the breast parenchyma immediately under the nipple, the second seed was positioned under the fascia of major pectoralis muscle, and the third seed was located in the breast gland in a plane equidistant as much as possible from the nipple and the muscular plane.

### Pre-operative and post-operative computed tomography (CT). Setup, simulation, and target definition

In our series, all patients were considered “suitable” or “cautionary” candidates as outlined in the European Society for Radiotherapy and Oncology (ESTRO) and American Society for Radiation Oncology (ASTRO) consensus statement for APBI [13, 14]. Prior to surgical treatment, patients also underwent a chest CT simulation in a supine position. CT scans were acquired with a thin layer (1.5 mm) from the base of the neck to the diaphragm.

A further chest CT-simulation was performed no earlier than 2 weeks, but within 4 weeks, after surgery. The setup procedure consisted in positioning the patient on a board with both arms along the body. In order to increase the angles available to the CyberKnife beams to aim at the tumor, the ipsilateral arm was distanced from the breast by elevating the patient’s chest from the mobile support of the CT scanner with 2 cm styrofoam boards. To avoid non-reproducible deformations, especially in the case of large breasts, no bras or other body shaping garments were used. Axial images of 1.5 mm slice thickness were acquired from the thyroid to the base of the lungs. The target volume on the basis of preoperative imaging was defined as the tumor bed. Based on evidence that 90% of tumor cells remaining after quadrantectomy are within 15 mm of the cavity edge, a safety margin of 1.5 cm was used to obtain the Clinical Target Volume (CTV). The Planning Target Volume (PTV) was defined as CTV plus an additional margin of 5 mm. Sometimes a smaller margin was used near the skin in order to keep a distance of 5 mm between skin and PTV. The heart, bilateral lungs, thyroid, skin and ipsi-lateral and contra-lateral breasts were separately contoured as “organs at risk.”

### Treatment planning

Treatment planning for each patient was generated with the CyberKnife Multiplan Treatment Planning System, generally 3 days before treatment. The three fiducials implanted during surgical operation were identified on the CT scan, in the three planar views, to define the target position during treatment delivery. The treatment plan was then optimized using an Iris collimator to deliver a multiple, non-isocentric, non-coplanar beam set. Doses of 30 Gray (Gy) in 5 consecutive fractions were prescribed to the isodose line encompassing 95% of the PTV, minimizing the occurrence of hot-spots (i.e., maximum point dose *D*max < 105–115% of prescribed dose). Using the linear quadratic model and assuming an α/β ratio of 3, this corresponds to a biologically effective dose of 54 Gy in standard fractionation. Dose constraints, based on the NSABP/RTOG B-39 protocol [17], are summarized in Table 2. Extra care was taken to ensure that ipsilateral breast and lung doses were kept as low as possible. Optimization was addressed to reduce both the mean lung dose (MLD) and mean heart dose (MHD) for ipsilateral lung and heart, as well as the mean dose to the skin and ipsilateral breast. During treatment planning, dose volume histogram analyses were conducted to determine whether dose constraint guidelines, as established by the NSABP/RTOG trial, could be met. The volume of ipsilateral breast that received 5%, 50%, 80%, and 100% of dose was recorded.

**Table 2:** Dose constrains

Organ at risk (OAR)	Volume	Dose (Gy)
Ipsi-lateral lung	< 10%	30% of the prescribed dose
Contra-lateral lung	< 10%	5% of the prescribed dose
Heart (right breast)	< 5%	5% of the prescribed dose
Heart (left breast)	< 40%	5% of the prescribed dose
Thyroid	Maximum point dose	≤3% of the prescribed dose
Skin	<10cm^3^	36.5 Gy
	Max point	39.5 Gy
Ipsi-lateral breast (PTV excluded)	< 40% < 20%	≥ 50% of the prescribed dose ≥ 100% of the prescribed dose
Contra-lateral breast	100%	< 3% of the prescribed dose

*PTV* Planning Target Volume

The treatment plan was delivered using the Synchrony Respiratory Tracking system for real-time image guidance. This system monitors the patient’s respiratory motion through Light Emitting Diodes, which are placed over the abdomen/thorax area with the maximum respiratory excursion and tracked continuously using a CCD camera. Contextually, the system acquires live images of the fiducials at user-defined intervals and calculates offsets based on digitally reconstructed radiographs. Using this information, the Synchrony system builds a model correlating the two motion patterns and sends compensation data to the robotic manipulator, synchronizing breathing guided beam delivery with the motion of the target. In this way, the treatment beam locks onto the tumor with pinpoint precision, eliminating the need for gating techniques or restrictive body frames.

### Follow-up, toxicity, cosmetic evaluation, and quality of life assessment

Follow-up for acute or late skin toxicity evaluation consisted of clinical examination at 1,3,6,9,12, and 24 months from the end of the CK-APBI. Follow-up for evidence of loco-regional or distant disease consisted of planned chest X-ray, liver and breast ultrasound examination and mammography performed every 12 months. Three months after the end of the radiotherapy, all patients underwent chest CT to investigate eventual radiation-induced lung injury.

Acute skin toxicity was scored at the end of CK-APBI up to 3 months, whereas late skin toxicity was considered starting from 6 months after treatment. The RTOG/EORTC toxicity scale was employed for acute and late effects and for scoring the maximal detected toxicity [31]. Skin toxicity considered the following endpoints: erythema, dry skin, edema, ulceration/hemorrhage, telangiectasia, fibrosis/induration, hyperpigmentation, and atrophy. Late skin toxicity and cosmesis are referred to the time of last examination (24 months).

Cosmetic results with photographs were assessed pre- and post-CK-APBI, and at 1,3,6,9,12, and 24 months based on the criteria of Harvard [32] defined according to 4 levels of evaluation: poor, fair, good, and excellent. Considering our limited sample size, we aggregated the two categories (fair and good) as a single intermediate category, and we defined three levels of evaluation: poor, fair/good, and excellent. Cosmetic results were independently evaluated by the patient, two physicians (a radiotherapist and a surgeon agree in evaluation), and other physician, as an external observer, not involved in the study.

### Statistical analysis

Numerical variables were summarized using mean, standard deviations (SD) or median, Inter-Quartile Range (IQR) as appropriate. Student’s t and chi-square tests were used to compare means proportions in two independent groups, respectively.

The association between two continuous variables was evaluated using Sperman’s rank correlation coefficient (ρ). Lin’s concordance correlation coefficient was used to estimate agreement between the evaluations of tumor size given by mammography, ultrasound examination, and magnetic resonance imaging [33].

The agreement between cosmetic evaluations performed by three observers (physician, patient, external observer) was assessed by Cohen’s Kappa. Magnitude guidelines for Cohen’s Kappa have been suggested by Landis and Koch [34]. The following values were proposed: < 0 indicating no agreement, 0–0.20 slight, 0.21–0.40 fair, 0.41–0.60 moderate, 0.61–0.80 substantial, and 0.81–1.00 almost perfect agreement.

## Results

From June 2013 to June 2014, 29 patients (median age 59, range 45–84 years) were considered eligible for the present study, of which 20 were evaluable. Nine patients were excluded according to previous criteria such as histology (invasive lobular carcinoma), multifocality shown at MRI, and pathological tumor size larger than 2 cm. Furthermore, one patient revoked informed consent, and two patients had breast and body size that are unsuitable for CyberKnife irradiation.

At radiological pre-surgical evaluation of the tumor, the mean size was 9.1 mm (SD ± 3.0, range 4.0–15.0), 9.6 mm (±3.4, 4.0–18.0), and 10.4 mm (±4.0, 5.0–18.0) by mammography, US, and MRI respectively. Evaluating Lin’s coefficient among these radiological examinations, mammography and US showed a value of 0.85 (confidence interval (CI) =0.73–0.97, *p* < 0.001), mammography and MRI of 0.50 (CI = 0.18–0.81, *p* = 0.002), and US and MRI of 0.60 (CI = 0.32–0.89, *p* < 0.001). Assuming pathological T size as a reference value (mean value 10.5 ± 4.3 mm, range 3.0–18.0), Lin’s concordance was 0.44 (CI = 0.12–0.76, *p* = 0.007), 0.59 (CI = 0.31–0.87, *p* < 0.001), and 0.76 (0.56–0.95, *p* < 0.001) for mammography, US and MRI, respectively.

All patients underwent quadrantectomy, whereas 19 of 20 patients had sentinel node biopsy. At histological examination, 18 of 20 patients had an invasive ductal carcinoma, and 16 patients showed a complementary smaller component of ductal carcinoma in situ (median 7.5%, IQR 5.0–17.5), consistent with the protocol indication. Fifteen patients had tumor grade as I-II, and only 3 and 4 patients had necrosis and microcalcifications, respectively, at histological examination. All patients were estrogen receptor positive, with 18 patients showing very high positivity, ranging from 66% to 100%. All but one patient were negative for HER2 receptor, whereas 14 patients (70%) had Ki-67 < 20%. Evaluating surrogate subtypes defined by the St. Gallen International Breast Cancer Conference [35], 8 patients were considered as Luminal A-like, 11 as Luminal B-like, and 1 as Luminal B-HER2 enriched.

In all patients no migration was observed in the position of the fiducials at the time of treatment with respect to the position identified on treatment planning CT. The time allowed for the fiducials to stabilize before CT acquisition could therefore be considered appropriate.

Using the CyberKnife system with Iris, treatment time including patient set-up on treatment couch was approximately 60 min, with a range from ~ 35 min to ~ 120 min. The longer time (120 min) was limited to the initial treatments and was a consequence of the inexperience of the team, especially in the patient setup and fiducial alignment phase. In general, larger breasts were associated with increased mobility, requiring longer patient set-up time. The team experience plays an important role on the treatment time that is comparable with other techniques using system for breathing management.

In two patients, inn some fractions, only two fiducials could be tracked reliably. This was due to the difficulty of finding a set of X-ray parameters producing a contrast image suitable for simultaneous identification of the three implanted fiducials. The occurrence of this problem was independent of patient size and was generally caused by the imaging system mistaking hyperdense or bony landmarks of the humerus or ribs for fiducials. The positional adjustment in case of a fiducial that was not identified automatically was always manually attempted by the radiation therapist; correspondence between the fiducial location on the live and planned images was subsequently inspected and confirmed by the radiation oncologist. An important aspect of CK-APBI tracking system is the possibility to manage the movement also with two markers. To overcome the migration of a fiducial could be to implant more than three markers, especially in the large breast. Moreover, the PTV margin was considered adequate to accommodate for possible residual tracking errors of the fiducials, producing safe and effective treatment.

All patients received the prescribed dose of 30 Gy in five fractions delivered to the PTV.

The median number of beams was 180 (IQR 107–213; range:56–325) with a median PTV isodose prescription of 86.0% (IQR 85.0–88.5; range:82–94). The median PTV was 88.1 cm3 (IQR 63.8–108.6; range:32.3–238.8). The median percentage of a whole breast reference volume receiving 100% and 50% of the prescribed dose (*V*100 = 30Gy and *V*50 = 15Gy) was 75.0% (IQR 52.5–104.0; range: 8.0–202.0) and 28.7% (IQR 20.0–32.6; range:13.0–57.0), respectively. The median PTV minimum dose was 26.2 Gy (IQR 24.7–27.6; range:22.3–29.3). Table 3 summarizes the median values with interquartile range for main radiotherapy treatment dosimetry and delivery.

**Table 3:** Radiotherapic treatment dosimetry and delivery

	Median (IQR)	Range
NCI	1.29 (1.19–1.52)	1.12–3.10
HI	1.16 (1.13–1.18)	1.06–1.25
Median PTV isodose prescription %	86.0 (85.0–88.5)	82.0–94.0
Total number of beams	180 (107–213)	56–325
Monitor Units	17,524 (13949–22,123)	7519–44,385
Volume of lesion (mm^3^)	88,098 (63835–108,617)	32,256–238,832
Homolateral lung V30%	2.3 (0.25–3.15)	0.0–9.4
Contralateral lung V5%	1.15 (0.00–3.65)	0.00–17.00
Heart V5% (right breast cancer)	0.2 (0.0–3.3)	0.0–5.2
Heart V5% (left breast cancer)	14.0 (12.1–29.7)	7.4–33.0
Thyroid Dmax (Gy)	0.26 (0.14–0.44)	0.09–1.00
Skin Dmax (Gy)	29.46 (27.97–30.33)	21.04–32.00
Homolateral breast V50%	28.7 (20.0–32.6)	13.0–57.0
Homolateral breast V100%	75.0 (52.5–104.0)	8.0–202.0
Contralateral breast D100% (Gy)	0.09 (0.03–0.12)	0.00–0.25
PTV Dmin (Gy)	26.2 (24.7–27.6)	22.3–29.3
PTV V90%	100 (98.7–100)	92.0–100

*NCI* normalized conformity index, *HI* homogeneity index, *Dmax* maximum dose, *Dmin* minimum dose, *Vx%* percentage of volume receiving x% of the prescribed dose, *Dx%* dose received by x% of the volume of interest

In one patient, the contralateral lung receiving 1.5 Gy was 17% (NSABP guidelines V1,7 < 15%). This was principally due to the thin built of the patient and the particular lateral localization in a adipose breast gland that was too close to the chest wall, preventing the creation of lateral oblique tangential beams. All other patients were kept well below than declared in our protocol as well as in the NSABP guidelines. Lastly, one patient received a 1 Gy maximum dose to the thyroid that was slightly higher than our established constraints, but which was otherwise lower than the NSABP/RTOG protocol (less than 1Gy).

Spearman’s correlation between the total number of radiation beams and total volume of the lesion was not statistically significant (ρ = 0.16, *p* = 0.500).

For evaluation of acute toxicity, cosmetic results, and medium term toxicity, patients were evaluated and results recorded immediately before and after radiotherapy (median time of control 5 days, IQR 4–6), and then after one (median 31 days, IQR 30–36), three (median 95 days, IQR 90–102), six (median 189 days, IQR 178–206), nine (median 280 days, IQR 270–303), 12 (median 367 days, IQR 356–408), and 24 months (median 734 days, IQR 688–765).

**Fig. 1:**
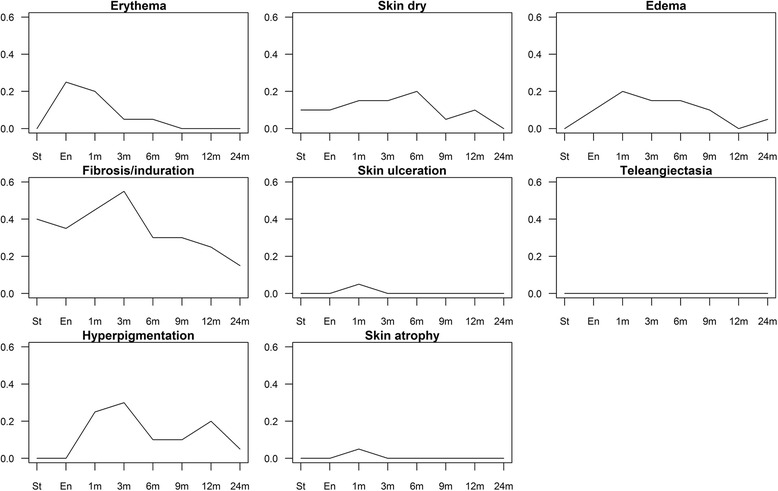
Prevalence of side effects in the study population for different time points

Mild side effects (almost all transient) were recorded during the period of observation, with no need for therapeutic intervention. The prevalence of side effects is shown in Fig. 1.

Regarding patients with erythema, recorded in at least one period of observation, the median skin volumes of absorbed doses (5,10,15,20 Gy) were statistically borderline significantly lower than those of patients without erythema [35.0 vs 47.1 (*p* = 0.104); 15.9 vs 25.7 (*p* = 0.065); 9.5 vs 17.7 (*p* = 0.051); 5.0 vs 11.7 (*p* = 0.065), respectively]. The number of observed periods with presence of erythema showed an inverse, moderate correlation both with volume of skin absorbing different doses (5,10,15,20 Gy) of radiation (Spearman’s correlation ρ ranging from − 0.49 to − 0.44, *p*-values ranging from 0.057 to 0.089), as well as with homolateral breast V50% parameter (Spearman’s correlation ρ = − 0.47; *p* = 0.036).

As far as fibrosis is concerned, the number of observed periods with presence of fibrosis positively correlated with PTV minimum dose (Gy) (Spearman’s correlation *ρ* = 0.45; *p* = 0.046).

No radiation-induced lung injury was recorded in any patient by chest CT performed at three months after the end of radiotherapy.

**Fig. 2:**
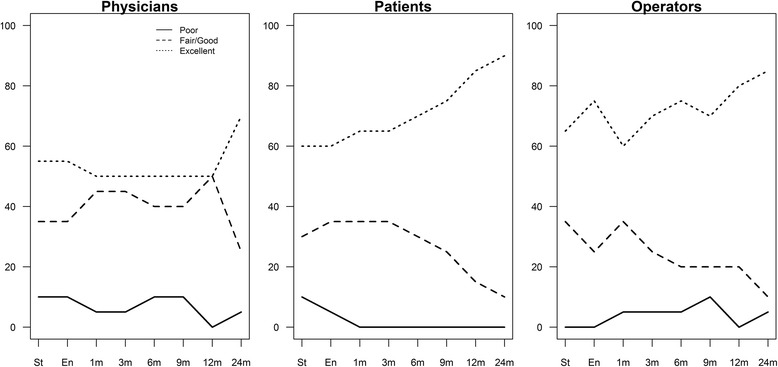
Percentage of “Poor”, “Good” and “Excellent” evaluations given by physicians, patients and operators

Cosmetic evaluations were performed by three different observers (physician, patient, external observer) at each control visit, from the start of radiotherapy to 2 years, as previously specified. Figure 2 shows the evaluations of the three observers, classified as poor, fair/good, and excellent. Specifically, patient evaluations progressively increased from 60% up to 85%,and this trend is similar to that of the external observer. After the first month, a poor rating was no longer recorded by patients. Conversely, the physicians’ excellent evaluation was different, constantly around 50%, but a poor rating was no given in more than 10% of cases during the entire period of observation.

The agreement among observers estimated by Cohen’s Kappa is summarized in Table 4. According to the Landis and Koch classification, the estimated agreement between patient and physician in the seven observation points ranged from moderate to slight, as well as between patient and external observer, whereas between physician and external observer the agreement ranged from fair to substantial.

**Table 4:** Inter-rate agreement between patient, physician and outside observer (O.O.) evaluations of cosmetic results at time of starting and ending of radiotherapy and after 1, 3, 6, 9, and 12 months; Cohen’s kappa with confidence interval and *p* value of the two-sided hypothesis test

	Physician - Patient	Physician – O.O.	Patient – O.O.
Start	0.37 (− 0.03–0.70) *P* = 0.019	0.23 (− 0.12–0.61) *P* = 0.113	0.41 (0.05–0.77) *P* = 0.016
End	0.45 (0.03–0.78) *P* = 0.008	0.20 (− 0.09–0.59) *P* = 0.133	0.57 (0.22–0.92) *P* = 0.002
1 month	0.42 (0.09–0.78) *P* = 0.018	0.63 (0.25–0.91) *P* < 0.001	0.59 (0.25–0.93) *P* = 0.002
3 month	0.42 (0.07–0.79) *P* = 0.018	0.63 (0.29–0.91) *P* < 0.001	0.56 (0.19–0.93) *P* = 0.003
6 month	0.15 (−0.18–0.60) *P* = 0.204	0.54 (0.22–0.89) *P* < 0.001	0.52 (0.13–0.91) *P* = 0.005
9 month	0.24 (− 0.06–0.60) *P* = 0.086	0.64 (0.34–0.91) *P* < 0.001	0.53 (0.16–0.90) *P* = 0.002
12 month	0.10 (−0.21–0.41) *P* = 0.266	0.40 (0.08–0.72) *P* = 0.013	0.14 (− 0.35–0.63) *P* = 0.266
24 month	0.28 (0.00–0.77) *P* = 0.051	0.60 (0.00–1.00) *P* < 0.001	0.56 (0.04–1.00) *P* = 0.001

Finally, after a median follow-up of 27.7 months (IQR 25.7–32.0), no local recurrences or distant relapses were recorded in our series.

## Discussion

Our pilot study showed the good feasibility and safety of CK-APBI in early breast cancer. As far as acute and sub-acute toxicity are concerned, we recorded only mild and almost all transient side effects during the period of observation, with no need for therapeutic intervention. Late cosmetic results were good with negligible toxicities.

During the 1970s, in the new era of breast conserving surgery for early breast cancer, the rational for WBRT, typically delivered in 5–6 weeks, was to eradicate any remaining cancer cells near to or distant from the surgical cavity.

The rationale of PBI/APBI, i.e. to restrict the radiotherapy target to the lumpectomy cavity in selected patients with low recurrence rate risk, is that the large majority of recurrences after BCS are close to the original tumor site [10, 11]. However, in a wide review of the main trials, PBI/APBI appeared to be significantly worse that WBRT (hazard ratio 1.74, *p* = 0.002) in terms of local-recurrent free survival; conversely, cosmesis appeared to improve and overall survival and disease-free survival were highly similar [36]. All this low-quality of evidence data need to be confirmed with the results of ongoing trials [36].

All other currently used APBI techniques show not-negligible drawbacks. The major disadvantages of IORT are the lack of final pathological results before delivering radiation and, when low-energy X-rays are used (as in the Targit study) [37] the potential for low therapeutic efficacy to the more distant margins of the target volume. Multicatheter interstitial brachytherapy, that represents one of the earliest modalities used for APBI and therefore has the longest follow up, is technically challenging, requires extensive operator training and limits its broad availability. The invasive nature of catheter placement may lead to acute complications as pain, infections and hematoma. Intracavitary brachytherapy utilizing balloon catheters is a less invasive procedure, but it may not be suitable for cavities close to the skin surface or for irregularly shaped cavities to which the balloon cannot conform. APBI delivered with three-dimensional conformal radiotherapy (3D-CRT) or intensity modulated radiotherapy (IMRT) technique is subject to intra-fractional and breathing motion, surface deformation and treatment set-up uncertainties that have to be accounted in the PTV. Large amounts of normal breast tissue may receive high-dose irradiation with an increased risk of poor cosmesis. CK-APBI offers technical improvements in partial breast irradiation using real-time tracking, respiratory motion management and submillimeter accuracy with few technical limitations. Reduced target and treatment uncertainty allows for treatment intensification, maximal target coverage while protecting normal tissue from unnecessary high-dose irradiation.

In response to the concerns and limitations related to breast radiotherapy techniques, the use of CK-APBI was conceived for early breast cancer [26], which involves a short course of intense treatment focused on the target tissue, with small margins for geometric uncertainty: sharp dose gradients enhance the need for high accuracy. To date, there is very little experience with stereotactic radiotherapy for breast cancer [26–30], although there is very limited body of literature on this topic, published data on dosimetry of CK-APBI are very encouraging [30, 38].

A few studies have compared differences in dose delivering among the different techniques for APBI currently available. All authors comparing CyberKnife versus other techniques, i.e. 3D-CRT, IMRT and tomotherapy, concluded that CyberKnife can achieve a highest level of PTV coverage and, generally, the deepest dose gradient with better dose sparing of organs at risk and, in particular, of the homolateral breast [27–29, 39]. Consequently, the involved tissue volume can be reduced by treatment with CyberKnife [27, 28]. Furthermore, the fiducial-based synchrony tracking method used by CyberKnife allows adding a minimal margin to CTV when creating PTV, allowing for greater spare of skin surface, chest wall, and pectoralis muscles. In general, we used a greater margin between CTV and PTV than that considered by Rault et al. [29]: however our PTV dimensions were consistent with other experiences [30, 38], but to reduce the skin dose we often applied a smaller margin near the skin to keep a margin of 5 mm between skin and PTV. Moreover, CyberKnife offers the possibility to fractionate the dose through a large number of beams (in our experience from 56 to 325) allowing distribution of the skin dose on a larger area, making the treatment more acceptable. This strategy was important to achieve good cosmetic outcomes, while reducing fibrosis as much as possible.

The increase in late toxicity is commonly considered one of the main disadvantages in APBI, especially considering cosmetic outcomes. Moreover, late toxicities are related to the dose delivered, in particular the dose per fraction and tissue volume involved.

In our study, with a follow up of 24 months, no patients experienced fair/poor cosmesis or fibrosis and good/excellent rates were recorded. Our good results, in term of acute and late toxicities, are also due to the dose delivered. The fractionation in our protocol was firstly adopted by Formenti [40] and in other few studies [26, 30, 40, 41] and all reported no acute toxicities and good cosmetic outcomes. On the contrary, Leonard et al [42] adopted a different fractionation in which 38.5 Gy was delivered in 10 fraction twice a day. Using this fractionation, they reported grade 2–4 and 3–4 fibrosis in 31% and 7.5% of patients, respectively, after a median follow-up of 32 months. In addition, they registered 11% of fat necrosis and 19% of fair/poor cosmetic outcomes. An interim report from a large randomized controlled trial found that the 3D-CRT increased rates of adverse cosmetic outcomes [43].

Cosmesis is dependent on the target volume [44] and consequently on the ipsilateral breast volume treated: Liss et al. reported poor outcomes in terms of fibrosis with partial breast IMRT when the mean percent of whole breast reference volume receiving 50% (V50) and 100% (V100) of the prescribed dose was greater than 46% and 23%, respectively [45].

Some possible causes may be the high volume of breast receiving 50% of the prescribed dose, as usually found with 3D-CRT or IMRT conformal or intensity modulated radiotherapy where the moving target beneath the fixed beams needs a large PTV to be accurately covered.

CyberKnife delivers a relatively steep dose gradient outside and within the target volume, mimicking brachytherapy dose distribution, and overcoming its invasive and challenging characteristics.

In our series, with a prescribed isodose line of 87%, V50 and V100 levels were kept to 28.7% and 10.7%, respectively, which are very similar that reported in published CyberKnife experiences [26, 30, 38, 39].

After a median 24 months follow-up, there were no recurrences in our group of patients. The correct selection of patients candidate for APBI, according to the ASTRO and ESTRO guidelines, may be a crucial point. From this point of view, instrumental radiologic evaluation is fundamental to avoid not considering some patients eligible due to extensive intraductal disease or multifocal tumors, not otherwise detected before final histology. To address this concern, we added breast MRI evaluation to standard mammographic and US examinations, considering MRI for its better ability to detect multifocal/multicenter disease and to have a further measurement of the lesion. Next, we tried to examine the added value of this additional pre-surgical evaluation. We used Linn’s concordance among these radiologic tools, and between each of these radiologic examinations pathologic size was considered as a reference parameter. Globally, the concordance is poor, except for the evaluation between mammography and ultrasound which showed moderate concordance, according to McBride agreement criteria [46]. Considering this poor concordance between radiological tools and tumor size, MRI was more precise than mammography and slightly better than US in defining the size of the lesion using the pathological measurement as a reference; however, the better ability of MRI and the error of mammography and US did not seem to be related to the presence and rate of the intraductal component of the disease. Conversely, the error of measurement of MRI was negatively correlated with tumor size.

Other crucial point is the intra-operative implantation of gold fiducials for optimal tracking of the motion of the breast during treatment via the CK Synchrony system. Goggin et al.*..* suggested that the spacing between each fiducial pair should be > 20 mm, and the angle between any fiducial should be > 15° for optimal tracking [28]. We substantially agree with this point of view, positioning three fiducials at the each apex of a theoretical triangle, but with the three fiducials sited on different planes. We believe that there may be two main problems: first the structure/size of mammary gland, in which adipose tissue may be sub-optimal in avoiding the migration of the fiducial to a different site and, secondly, the possibility to standardize the criteria used to position these fiducials. We believe that positioning the fiducials in the glandular parenchyma under the main ducts of the nipple and under the muscular fascia of pectoralis major may be stable and well-recognized sites and planes. Only the third fiducial has to be positioned by identifying an isle of glandular parenchyma in a plane between the previous two planes.

Interestingly, Rahimi et al recently studied the tolerability of CK-APBI, evaluating a dose escalation approach up to 40 Gy delivered in 5 fractions [47]; but, assuming that a 30 Gy total dose in 5 fractions is equivalent to 54 Gy in a standard 2 Gy fractionation [41], we believe that this level of dose is sufficient for selected low risk early breast cancer.

With the technical advantages over existing PBI platforms are evident, potential disadvantages exist with CK-APBI. We are aware of the high cost of CK, especially if compared with IORT that, to date, is the most cost effective APBI option [48]. A more accurate cost analysis will be subject to future evaluations: the impact of such a sophisticated technique in protecting normal tissues from unnecessary high-dose irradiation, and consequently lowering toxicities, and its power in treatment intensification, with maximal target coverage and potential reduction of local recurrences may equal the initial costs for the health system.

## Conclusion

This study, although limited by the small number of patients and short duration of follow-up, suggests that CK-APBI is a feasible, non-invasive radiation technique in carefully selected patients. Our results are in agreement with the few published studies in terms of skin toxicity and cosmetic results. Starting from these encouraging results, we intend to perform CK-APBI in a larger series of breast cancer patients. Furthermore, longer follow-up will allow for better evaluation of long-term outcomes.

## Abbreviations

3D-CRT: Three dimensional conformal radiotherapy
APBI: Accelerated partial breast irradiation
ASTRO: American Society for Radiation Oncology
BCS: Breast-conserving surgery
CCD: Charge-Coupled Device
CI: Confidence interval
CK-APBI: CyberKnife radiosurgery system
CT: Computed tomography
CTV: Clinical Target Volume
ESTRO: European Society for Radiotherapy and Oncology
Gy: Gray
IMRT: Intensity modulated radiotherapy
IOERT: Electron Intra Operative Radiation Therapy
IORT: Intraoperative radiotherapy
IQR: Interquartile range
MHD: Mean heart dose
MLD: Mean lung dose
MRI: Magnetic resonance imaging
PBI: Partial breast irradiation
PTV: Planning Target Volume
SD: Standard deviation
US: Ultrasound
V50 and V100: The mean percent of whole breast reference volume receiving 50% (V50) and 100% (V100) of the prescribed dose
WBRT: Whole breast radiotherapy
